# Conditions Where Internal Drugs Are Indicated

**Published:** 1904-03

**Authors:** J. P. Buckley


### CONDITIONS WHERE INTERNAL DRUGS ARE INDICATED.

We will now consider a few pathological conditions, often confronting the dentist, where internal drugs are indicated.

Take, for instance, a putrescent pulp where some of the infectious material has been forced through the apex of the root by virtue of the gases generated in the decomposition, by instrumentation or otherwise, and an abscess is developing, which is characterized by that dull, dead, gnawing, grinding pain, produced by the pus trying to bore through the alvolus; that condition where the dentist knows, or should know, that counter-irritation gives but slight or temporary relief, in such instances as this we do not deserve the confidence placed in us by our patients, should we dismiss them without prescribing a general hypnotic, even if morphine must be given to control the pain. A prescription, however, should never be written for morphine, as the patient, being satisfied with the effect produced, could get the prescription refilled on the least provocation, and might, perchance, innocently acquire the habit. But in this case morphine could be given and the patient not know what drug had been taken. Now I wish to be understood as not advocating the injudicious, indiscriminate, or miscellaneous use of such drugs as morphine; but I do desire to say that where the drug is indicated it should be administered; and it seems to me that there is no condition where the effect of morphine is more beneficial, if judiciously used, than in the case to which I have called attention. There are other drugs that can be given with marked benefit, such as codeine (a milder alkaloid of opium), and many of the coal-tar preparations are valuable, among which I might mention phenacetine, acetanilid, etc. But opium, especially its chief alkaloid, morphine, is the one drug, par excellence, to stop pain, as this drug acts upon and controls the central nervous system. Alterative drugs are also indicated here. The best and most easily administered is potassium iodide, prescriped with some of the elixirs, syrups, or waters, to mask the taste.

Another condition demanding internal drugs is that of facial neuralgia, especially where we are unable to, at once, locate and remove the cause. How often do we find the facial, or seventh cranial nerve of patients, expressing untold agony as the result of some disturbance of the trigeminus or fifth; and do our best, we are unable, at the time, to locate the cause of the disturbance. Now, what shall we do in this case? Who wants to admit that they are unable to give relief? Shall we acknowledge our ignorance of a subject that we are supposed to know and turn the patient over to the physician, when the disturbance is purely dental, and let the physician do what every dentist should have done—administer a drug internally to control the pain until the cause can be located and removed? No, let pathological conditions, of dental origin, be treated by dental surgeons; and if the latter feels unable to treat these cases, then he should study his materia medica. I admit that we are sadly in need of good modern text-books on dental materia medica, but such a text-book would be more helpful to the dental student than to the dental practitioner. All general text-books on this subject are for dentists as well as for other specialists of medicine. The drugs indicated here are the hypnotics. Again I must say that there is no drug that will serve us better than morphine, if intelligently administered, in these severe neuralgic conditions where we are unable to locate the cause and must control the pain.—*Dr. J. P. Buckley, Ex. Summary.*

